# Biomarkers in nutrition: new frontiers in research and application

**DOI:** 10.1111/nyas.12069

**Published:** 2013-03-11

**Authors:** Gerald F Combs, Paula R Trumbo, Michelle C McKinley, John Milner, Stephanie Studenski, Takeshi Kimura, Steven M Watkins, Daniel J Raiten

**Affiliations:** 1 Grand Forks Human Nutrition Research Center, U.S. Department of Agriculture, Agricultural Research ServiceGrand Forks, North Dakota; 2 Center for Food Safety and Applied Nutrition, U.S. Food and Drug AdministrationRockville, Maryland; 3 School of Medicine, Dentistry and Biomedical Sciences, Queen's University BelfastBelfast, Northern Ireland, United Kingdom; 4 National Cancer Institute, National Institutes of Health, U.S. Department of Health and Human ServicesRockville, Maryland; 5 University of PittsburghPittsburgh, Pennsylvania; 6 Ajinomoto Co., Inc.Tokyo, Japan; 7 Tethys Bioscience, Inc.Emeryville, Dalifornia; 8 Eunice Kennedy Shriver National Institute of Child Health and Human Development, National Institutes of Health, U.S. Department of Health and Human ServicesRockville, Maryland

**Keywords:** biomarkers, nutrition, nutrients

## Abstract

Nutritional biomarkers—biochemical, functional, or clinical indices of nutrient intake, status, or functional effects—are needed to support evidence-based clinical guidance and effective health programs and policies related to food, nutrition, and health. Such indices can reveal information about biological or physiological responses to dietary behavior or pathogenic processes, and can be used to monitor responses to therapeutic interventions and to provide information on interindividual differences in response to diet and nutrition. Many nutritional biomarkers are available; yet there has been no formal mechanism to establish consensus regarding the optimal biomarkers for particular nutrients and applications.

Sponsored by the Sackler Institute for Nutrition Science and the New York Academy of Sciences, the conference “Biomarkers in Nutrition: New Frontiers in Research and Application” was held on April 18, 2012 at the New York Academy of Sciences in New York City. The meeting, comprising individual talks and group discussions, brought together scientists and practitioners from industry, academia, and governmental organizations to discuss the current state of knowledge about nutritional biomarkers, to identify important challenges and unanswered questions, and to catalyze new research toward the common goal of implementing nutritional biomarkers in a broad, cost-effective, and meaningful way.

## The Biomarkers of Nutrition for Development (BOND) program

Gerald F. Combs, Jr. (USDA Grand Forks Human Nutrition Research Center) spoke on behalf of Daniel J. Raiten (Eunice Kennedy Shriver National Institute of Child Health and Human Development, NICHD) about the BOND Program. Supported by the Bill and Melinda Gates Foundation and managed by NICHD, BOND aims to harmonize the discovery, development, and distribution of biomarkers of nutritional status and to provide advice to researchers, clinicians, and policymakers on how best to use nutrition biomarkers.

Food and nutrition play key roles in supporting health and preventing disease. Globally, maternal and child undernutrition results in some 3.5 million deaths annually, accounting for 35% of the disease burden in children under five years of age.^1^ Undernutrition includes what has been called *hidden hunger*, that is, single and multiple micronutrient insufficiencies that affect two billion individuals in both industrialized and developing countries,^2^ and in those overweight or underweight.^3^ At the same time, overweight and obesity are becoming more prevalent, with an estimated one billion adults and 22 million children being overweight.[Bibr b4] Thus, the dual burden of over- and undernutrition presents a major challenge.^5^

It has been noted that the ability to assess the health impacts of nutritional status depends on the availability of accurate and reliable biomarkers that reflect nutrient exposure, status, and effect.^1^ Biomarkers are essential in this regard; yet, confusion remains surrounding their use and application. What might be a useful index of nutrient exposure may not necessarily reflect nutrient status, which, in turn, may not necessarily reflect the impact or function of that nutrient. Systematic reviews of a range of nutritional biomarkers have emphasized the lack of clarity in the definition of biomarkers and their application and purpose.^6^ The usefulness of even the most well-documented biomarkers has been limited by gaps in the understanding of their physiologic significance.

The BOND program was created to address this need; it is supported by a consortium that includes the Bill and Melinda Gates Foundation (BMGF), PepsiCo, the NIH Office of Dietary Supplements, and the NIH Division of Nutrition Research Coordination, and includes memberships with organizations and agencies representing the breadth of the global food and nutrition community. BOND is managed by the NICHD and aims to harmonize the process of making decisions about the best uses of biomarkers in individual situations.

BOND has targeted four primary user communities for its translational activities:

1. research (including basic research examining the role of nutrition in biological systems, clinical research, and operations research);
2. clinical care;
3. programs (surveillance to identify populations at risk, and monitoring and evaluation of public health programs); and,
4. policy (evaluation of the evidence base to make national or global policy about diet and health, and funding agencies that make decisions about priorities in food and nutrition).

Biomarker needs are, therefore, both general and user specific.

The BOND program was initiated through a consultative process with the food and nutrition community that culminated in an organizational conference held in Vienna in 2010, organized by NICHD and hosted by the International Atomic Energy Agency. Partners included key multilateral U.S. agencies and public and private organizations. That assembly endorsed the need to develop a process to inform the community about the relative strengths/weaknesses and specific applications of various biomarkers under defined conditions. Specific attention was paid to the needs for nutritional biomarkers in four use areas: research, clinical, policy, and programs. Five micronutrients of public health importance (iron, zinc, vitamin A, folate, and vitamin B_12_) were discussed as case studies with respect to new frontiers in science and technology. An overview of that meeting was published.^7^

The mission of BOND was developed in the Vienna meeting and included (1) developing consensus on accurate assessment methodologies relevant to users domestically and internationally, and (2) providing evidence-based advice to support a range of activities of the entire food/nutrition research and global health enterprise including (a) further development of national nutrition surveys, (b) review and development of dietary guidance, (c) development of new and improved systems of food/nutrient delivery, (d) monitoring and evaluation of new and existing programs and interventions, and (e) basic and clinical research to generate new data on diet and disease relationships and the roles of nutrients in promoting health and preventing disease.

**Figure 1:**
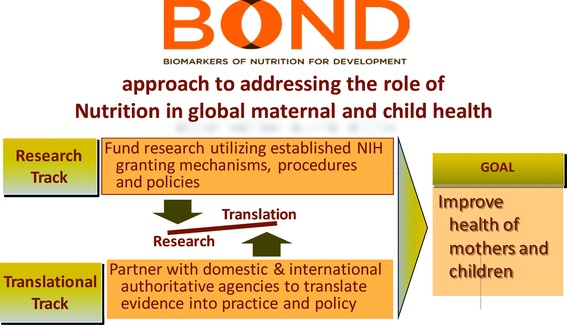
The Biomarkers of Nutrition and Development (BOND) program.

BOND is implementing this mission through two approaches: a translational track involving partnering with U.S. and international agencies, and the development of a research agenda that will lead to funding opportunities supported by agencies and organizations across the breadth of the global research funding enterprise (Fig. 1).

After the Vienna meeting, NICHD received core funding from the BMGF to begin the BOND project. The initial phase of that project includes the establishment of an expert panel for each of the above-mentioned case nutrients plus iodine. These panels are charged with reviewing the relevant literature supporting decision points regarding specific biomarkers, the needs of specific user groups, opportunities for new technologies, and key knowledge gaps. In 2013, BOND will convene a meeting at which the panels will provide input for the development of queries and responses that reflect the primary user communities.

Combs concluded by pointing out that BOND has established a website (http://www.nichd.nih. gov/global_nutrition/programs/bond/) with links to member agencies and organizations, opportunities to provide input, content on biomarkers relative to specific nutrients, and overviews of cross-cutting issues relative to nutritional biomarkers. Ultimately, the website will house a query-based system enabling users to gain information on particular biomarker applications.

## Use of biomarkers to substantiate health claims

Paula Trumbo (U.S. Food and Drug Administration, (FDA)) described the process by which the FDA evaluates the scientific evidence for the use of biomarkers to substantiate health claims on the labels of foods and dietary supplements. She pointed out that, as part of its evidence-based systemic review, the FDA relies on surrogate endpoints (qualified risk biomarkers) in the premarket scientific review of health claims used for labeling foods and dietary supplements. Health claims provide information about the relationship between a food or food component and risk of a disease or health-related condition (e.g., a surrogate endpoint of disease risk). The review of scientific evidence involves the identification, classification, and rating of relevant studies; the evaluation of the strength of the evidence; and the determination of whether that evidence supports a health claim.

**Figure 2:**
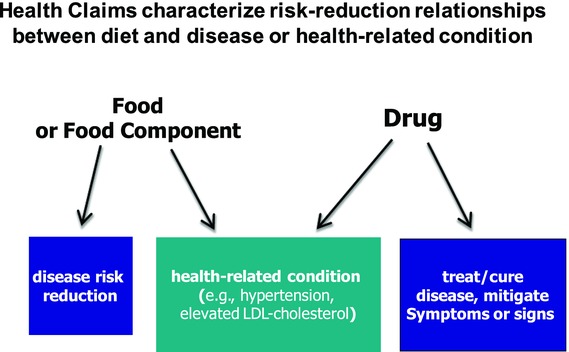
Risk reduction relationships implied by FDA-approved health claims.

The FDA relies on a limited number of available surrogate endpoints for its health claim reviews. For this reason, the FDA funded the Institute of Medicine (IOM) to develop a framework for the qualification of risk biomarkers. That report^8^ emphasizes the need for validated analytical methods that can measure risk biomarkers. The recommended qualification framework also calls for evaluating the relationship between the risk biomarker and the clinical endpoint (Fig. 2), as well as the need for evidence that the intervention affecting the risk biomarker also influences the clinical endpoint. This report is being considered by the FDA for future risk biomarker qualification.

Trumbo concluded by pointing out that the FDA Center for Drug Evaluation and Research manages a biomarker qualification program that includes the review of biomarkers of chronic disease risk. Such a program could possibly evaluate biomarkers that are applicable to health claims.

## Biomarkers of selenium status

**Figure 3:**
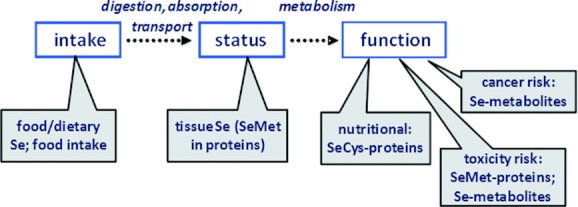
Types of biomarkers available for assessing Se intake, status, and function.

Gerald Combs reviewed the use and interpretation of biomarkers of selenium (Se) status in light of the current understanding of Se metabolism. Status with respect to the essential nutrient Se is considered under four categories relevant to human nutrition and health: (1) assessment of Se intake/exposure, (2) assessment of risk of nutritional Se deficiency, (3) assessment of Se adequacy for cancer risk reduction, and (4) assessment of risk of Se toxicity (Fig. 3). He pointed out that each category relies on a different set of endpoints with different evidence bases.

The nutritional functions of Se appear to be discharged by a group of selenoproteins, the best characterized of which (glutathione peroxidases, selenoprotein P) can serve as biomarkers of Se status. Some 25 selenoproteins have been identified; each can incorporate Se from inorganic Se compounds (selenite, selenate) and the amino acid analog selenocysteine (SeCys), but not from the dominant food form selenomethionine (SeMet), although the latter can freely replace methionine in protein biosynthesis.

This means that Se intake and exposure can be assessed on the basis of the Se contents of accessible specimens (e.g., plasma, urine, hair/nails, and buccal cells) if the dominant form of ingested Se is known. The Se contents of these tissues reflect the rate of intake of inorganic forms or SeCys only to the point of maximal selenoprotein expression, which occurs with approximately 40–50 μg Se per day or plasma Se levels of 70–80 ng/mL. If, however, Se is consumed in the form of SeMet, tissue levels can increase over a virtually unlimited range, reflecting the nonspecific incorporation of the element into proteins.^9^,^10^ While such cases can be assessed for Se exposure, they offer no information about function.

The potential for Se to reduce cancer risk is being actively researched. Current evidence suggests that risk reduction occurs among those with nutritionally adequate (i.e., maximal selenoprotein expression), but not high, Se status. For example, the Nutritional Prevention of Cancer Trial^11^ found that supplemental Se reduced cancer risk in nondeficient Americans with baseline plasma Se levels <120 ng/mL. This conclusion is consistent with the results of the larger SELECT trial.^12^ Thus, it appears that candidates for Se protection against cancer can be identified using Se biomarkers similar to those used to assess Se exposure, but with the application of a great target plasma level.

The potential for very high Se status to produce adverse physiological effects has been established from animal studies and accidental exposure on humans; these physiological effects have produced an array of clinical indicators but few biomarkers with predictive potential. For this reason, the default choice has been to use the highest Se tissue levels observed with no adverse effects as risk indicators. Some studies show no adverse effects with plasma Se levels <1000 ng/mL; however, recent studies have suggested that plasma levels >140 ng/mL may increase type 2 diabetes risk.^13^,^14^

Combs concluded by reiterating the two questions dominating the consideration of health roles of Se: Who may benefit from increasing Se intake? and Who may be at risk from increasing Se intake? He pointed to the need for better biomarkers of Se function to address each of these questions.

## Rationale and process for developing biomarkers for sarcopenia

Stephanie Studenski (University of Pittsburgh) discussed the use of biomarkers in the study of sarcopenia. Losses of muscle mass and strength seem to be almost universal age-related phenomena and are associated in epidemiological studies with numerous adverse outcomes including disability and mortality.^15^ She pointed out that, in order to develop useful biomarkers, such conditions must be clearly defined. While several definitions have been proposed for sarcopenia, which generally indicates loss of muscle mass, challenges remain. First, the criteria for low muscle mass were initially developed using sample population distributions and without considering the effect of strength. Second, more recently proposed definitions have been based only on expert opinion without a formal evidence base.

In order to address these challenges, the Biomakers Consortium was funded through the Foundation for the NationaI Institutes of Health, with the goal of pooling data related to body composition, muscle strength, functional abilities, and other relevant factors from multiple longitudinal and clinical trial studies of older adults. The model used by the consortium, as well as prior expert panels, suggests that the underlying clinical process proceeds from abnormal muscle mass or quality to muscle weakness, which results in reduced physical function and disability. In order to apply this model within a clinical diagnostic framework, the consortium suggested that older people would present clinically with complaints of reduced functional abilities that could then be assessed objectively by clinicians using physical performance tests, such as walking speed or ability to rise from a chair. Accordingly, the specific aims of the pooled analyses were to (1) determine criteria for clinically important weakness based on optimal discrimination between older persons with and without reduced physical performance; (2) determine criteria for clinically important low muscle mass based on the optimal discrimination between older persons with and without criteria for clinically important weakness; and (3) assess longitudinally whether the criteria defined in the first two aims help predict the onset of future physical disability.

The final pooled sample of older adults encompasses over 30,000 individuals of diverse age, gender, ethnicity, function, strength, and body composition. All major analyses have been completed and were presented at a conference in May 2012 to an audience of clinicians, regulators, scientists, and representatives from the private sector. There was general agreement that a clinical definition must include both weakness and low muscle mass, and that physical performance measures were the optimal choice for primary outcome measures because they are objective, reliable, and clinically relevant markers of function. There was also general agreement that older persons may be weak for reasons other than low muscle mass, because the muscle is not producing adequate force due to factors either within or outside the muscle itself. Therefore, it was suggested that poor muscle quality be used to define persons who are weak but who do not have low muscle mass.

In order to further evaluate the causes and treatment of poor muscle quality, the key next step is to develop criteria for inadequate force production per unit of muscle mass. Potential contributors to poor muscle quality might include factors related to muscle composition (including proteins and lipids), cellular energetics, and neuromuscular control.^16^

**Figure 4:**
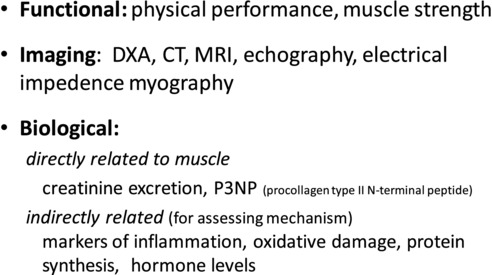
Candidate biomarkers of sarcopenia.

The development of biomarkers for sarcopenia is of particular value in the examination of mechanisms, diagnosis, and responses to treatment. A biomarker can be considered a characteristic that is objectively measured and evaluated as an indicator of normal biological processes, pathogenic processes, or pharmacologic responses to a therapeutic intervention. Biomarkers for sarcopenia might be grouped into three main types—functional, imaging, and biological—and could be used for screening, diagnosis, or endpoint assessment (Fig. 4). Among functional measures, objective indicators of physical performance might be useful for all three purposes. Measures of body composition, including muscle and fat, can be obtained from DXA, CT, MRI, or other techniques. While DXA is considered to be more widely available clinically and to have minimal respondent burden, there are substantial concerns about its ability to account for fat in muscle, while MRI and CT are superior for such detailed assessment of muscle but are more expensive and somewhat more burdensome. Therefore, some investigators have proposed using DXA for screening and CT or MRI for baseline and endpoint assessment. There are also newer techniques in development that are based on echomyography or electrical impedence myography. Most imaging techniques suffer to some extent from difficulty discriminating lean mass from water, so that some degree of error will be present in older persons with edema or other instances of excess body water.

**Figure 5:**
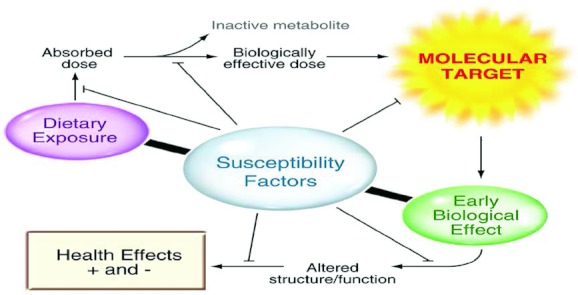
Need for biomarkers of host susceptibility in assessing the health impacts of bioactive substances.

**Figure 6:**
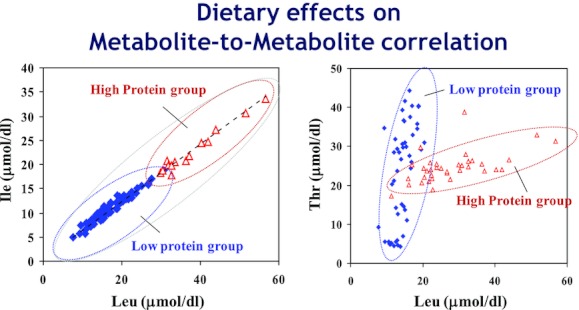
Opportunities to use disruptions in normal correlations among circulating free amino acids as biomarkers of health conditions, for example, effects of changes in dietary protein intake in the rat.

Studenski concluded by stating that new biological markers are needed to assess aspects of muscle metabolism directly or to assess other factors that influence muscle synthesis and degradation. Candidates of the former type include serum or urinary creatinine, creatine phosphokinase, or P3NP (procollagen type II N-terminal peptide). Those of the latter type include indicators of inflammation, oxidative damage, hormone levels, or protein synthesis.^17^ She noted that studies to assess the test characteristics of such biological indicators for sarcopenia are being implemented and that clinical trials for the treatment or prevention of sarcopenia, based on exercise, nutrition, and pharmacological approaches, are currently in the field or are being designed.

## Markers of dietary intake and risk of cancer

John Milner (National Cancer Institute (MCI) and National Institutes of Health (NIH)) described the myriad challenges and considerations involved in the development of sensitive and reliable biomarkers of dietary intake and cancer risk. He pointed out the increasing concern about noncommunicable diseases, especially cancer and heart disease, which centers on the lost productivity of individuals and the projected massive medical expense. It is abundantly clear that the increase in risk of cancer as well as heart disease is occurring in both developed and developing parts of the world. While part of the increase surely relates to an aging society and to the increased incidence of obesity, it is also becoming increasingly apparent that nutritional inadequacies are also contributors.

In order to evaluate the benefits of foods for health promotion and disease prevention, there is recognition that three types of biomarkers will be needed for an appropriate assessment. The first is a biomarker that evaluates intake or exposure. Unfortunately, the present methodologies are woefully inadequate and have large error terms, and thus may account for part of the wide variation in risk of noncommunicable diseases observed nationally and internationally. Not all fruits and vegetables are identical, and thus there is a need to identify which foods offer the greatest benefits for health promotion.

The second class of biomarker relates to the site of action (molecular target). Unfortunately, there is likely more than one target for bioactive food components, and thus unravelling the specific site of action of a bioactive food component is extremely challenging. This area is also complicated since normal and aberrant cell metabolism can influence the specific targets and thereby influence the response to bioactive food components. Since several foods may influence the same target, knowledge about dietary patterns will require increasing attention to explain the means to achieving maximum health benefits from the food and supplements that are consumed.

The third type of biomarker encompasses the interactions food components exhibit either among each other or with a person's genomic profile (Fig. 5). It is becoming increasingly recognized that genetics (polymorphisms, deletions, insertions, and copy number) and epigenetics (DNA methylation, histone homeostasis, noncoding RNA, and polycomb organization), along with transcriptome regulation, are critical to the response to foods and their components. These genomic, or more precisely nutrigenomic, factors influence multiple cellular processes that are reflected by shifts in specific proteins and cellular metabolites. Thus, proteomics and metabolomics are important technologies for assessing the impact of foods and their constituents at the phenotypic level.

Even these three biomarker categories are influenced by a host of internal and external insults that influence the needs for specific food components. Notable among these are the influence of excess calories (obesity), viruses, bacteria, and environmental contaminants. Thus, biomarkers are also needed to not only assess the omics of normal nutrition, but also the insults that can influence requirements under abnormal or diseased conditions. Finally, it must be recognized that many bioactive food components have the same site of action, and thus a much greater understanding of these interactions are needed to truly assess the benefits or risks in changing a person's diet.

Assessments of dietary intake and risk of cancer must also take into account the potential deleterious effects of modifying intakes, especially overindulgence. Increasing evidence points to harmful effects of consuming excessive amounts of specific foods or specific dietary supplements. These ill consequences can manifest themselves in multiple ways, including possible increases in cancer risk. It is unclear why this increased risk occurs. However, it may reflect the ability of excess amounts of specific bioactive food constituents to influence a metabolic pathway that is not normally influenced, or to create new and possibly deleterious metabolites. Either way, there is a need to identify individuals or subpopulations vulnerable to certain foods and their constituents. This is becoming a greater concern, given the large percentage of the population that consumes dietary supplements with the increased likelihood of excessive exposure.

Milner concluded that it is becoming increasingly clear that phenotypic changes can no longer be relied upon to identify benefits and risks associated with consuming specific foods or their components. The newest omic technologies must be embraced to identify those individuals who will benefit most from, or be placed at risk as a result of, dietary change. For this reason, he noted that intersectoral partnerships that build on academic, industrial, and governmental research strengths are critical for advancing knowledge about the relationships between diet and health.

## Blood-based markers of nutrient status and their association with metabolic risk

Steven Watkins (Tethys Bioscience, Inc.) discussed the fact that nutritional approaches can often be difficult to employ as prevention strategies in primary care settings, and that it is hard to argue that we are winning the battle against chronic disease. Yet, many large clinical studies have demonstrated that there is a profound effect of nutrients and lifestyle on the prevention of metabolic and cardiovascular disease.

One strategy for improving compliance with nutrition guidance is the enhancing of education for doctors and patients. Watkins suggested, however, that there may be another strategy more suited for use in a medical setting. Medicine in general is geared toward a treat-to-target approach, where diagnostics provide information on a patient's current status and whether corrective actions have made a positive impact. Nutrition is not enabled in this way, in that there is no objective score that can be used by clinicians as part of the treatment conversation with patients. While it is possible that physicians and patients can discuss diet records, it is a cumbersome method that still fails to capture metabolic individuality. Thus, nutrition remains focused on global recommendations rather than treat-to-target individualized advice.

Watkins discussed the development of assays to quantify the blood levels of many important dietary nutrients, including fatty acids, sterols, amino acids, and other markers of nutrient status, including acylcarnitines and bile acids. These assays have been used to identify the relationship of blood measures of nutrients with metabolic outcomes including conversion to diabetes. The concept is that by measuring the blood levels of key nutrients directly, patients and physicians can have simpler, more accurate and productive discussions about nutrition therapy. Nutrition advice that is individualized and targeted, as opposed to global, can be given.

Watkins described results of this approach using baseline samples from the Insulin Resistance Atherosclerosis Study,^18^ where they determined the association of baseline nutrient levels with risk for diabetes within five years. The study found strong positive associations of blood saturated fats and cholesterol synthesis intermediates, and strong negative associations between plant based fatty acids and sterols with diabetes risk. These observations are consistent with the nutrition guidance for diabetes prevention based on major clinical prevention trials.^19^ Additionally, weak positive associations between branched chain amino acids and odd-chain acylcarnitines and diabetes risk were observed.

These results suggest that using a measurement strategy, as opposed to diet recall, could enable a personalized approach to nutrition, where individual needs can be measured and nutrient advice can be dispensed in a treat-to-target paradigm instead of as global recommendations.

## Amino acid–based biomarkers for indicating nutritional and disease states

Takeshi Kimura (Ajinomoto Co., Inc., Japan) presented his group's innovative use of plasma amino acid profiles to screen for cancer and moderate malnutrition. He pointed out that, traditionally, biomarkers have typically been single molecules, the behaviors of which were pertinent to a phenotype or physiological state of interest. Modern metabolomic technologies, however, have made it possible to determine multiple metabolites and, thus, to facilitate capturing the status of specific biochemical pathways, offering unprecedented potential for generating biomarkers for various physiological states. Biomarkers generated in this manner have become important diagnostic criteria in various clinical areas; however, to date only a very small part of the information contained in the human metabolome has been used in the human health field.

Correlations of amino acid concentrations in various tissues have demonstrated that plasma amino acid patterns can serve as biomarkers for a number of diseases and physiological states (Fig. 6). The generation of such metabolomics data, however, requires that procedures for collecting, handling, processing, and analyzing samples be validated and standardized.^20^,^21^

Kimura noted that, after much testing, his group has developed a technology package to generate plasma amino acid–based markers for use in clinical settings. Their approach allows a single measurement of plasma amino acids to provide data on multiple biomarkers, each biomarker being a different amino acid profile. Both discriminative and surrogate markers can be generated depending on the target phenotype. Studies in rodent models indicate that this approach can also be used to distinguish protein malnutrition. Their approach has been used to generate biomarkers of risk for cancers of the stomach, lung, colon/rectum, prostate, and breast.^22^ Research is ongoing to determine whether specific plasma amino acid profiles can be used as biomarkers for hepatitis, irritable bowel syndrome, and metabolic syndrome.^23^,^24^

Kimura concluded by noting that, in April 2011, their technology was offered as a service to hospitals and clinics. As of July 2012, some 300 hospitals and clinics had adopted it as a blood test offered as an option to patients and healthy individuals.

## Biomarkers of fruit and vegetable intake

Michelle C. McKinley (Queen's University, Belfast) reviewed ongoing work to identify blood-based biomarkers of fruit and vegetable consumption. Much of the evidence relating food and nutrient intake to chronic disease risk relies on information gathered by various dietary assessment techniques. Such techniques are prone to random and systematic measurement errors, which may attenuate observed diet–disease associations.^25^ Therefore, biochemical biomarkers of dietary exposure are of great interest, as their use may improve the ranking of subjects for exposure to a particular food group or nutrient. Nutritional biomarkers also offer the possibility of an objective indicator of compliance with a particular dietary regimen in randomized controlled trials investigating the health effects of dietary modifications.

A systematic review of biomarkers of fruit and vegetable intake in human intervention studies^26^ included over 90 intervention trials. This included studies of three types: (1) whole-diet intervention studies (advice to increase fruit and vegetable intake was one component of a whole diet approach); (2) mixed fruit and vegetable studies (interventions involving administration of more than one type of fruit or vegetable); and (3) individual fruit and vegetable intervention studies (involving increased consumption of a certain type of fruit or vegetable).

These studies showed that a panel of biomarkers (α- and β-carotene, vitamin C, lutein, zeaxanthin, and β-cryptoxanthin) had value as indicators of compliance in fruit and vegetable intervention trials (Table 1). With the possible exception of fruit-only intervention studies, where assessment of vitamin C status alone may suffice, it seems rarely possible to rely on assessment of a single biomarker as an indicator of change in fruit and vegetable intake. The review also pointed to the need for more dose–response data to elucidate the natures of the dose–response relationships of specific biomarkers.

**Table 1:** Results of clinical intervention trials with fruits and vegetables, showing the value of biomarkers as indicators of compliance^26^

	Whole-diet studies (*n* = 11)		Mixed fruit/vegetable studies using counseling (*n* = 16)		Mixed fruit/vegetable studies using food provision (*n* = 36)	
			
	Measured	Reported increase	Measured	Reported increase	Measured	Reported increase
Biomarker	biomaker	(% use biomarker)	biomaker	(% use biomarker)	biomaker	(% use biomarker)
α-Carotene	7	3 (43)	14	12 (86)	21	16 (76)
β-Carotene	7	4 (57)	15	13 (87)	24	19 (79)
Lycopene	7	2 (29)	12	4 (33)	20	8 (40)
β-Cryptoxanthin	7	1 (14)	12	8 (67)	20	12 (60)
Lutein	4	3 (75)	7	5 (71)	16	11 (69)
Zeaxanthin	2	1 (50)	5	1 (20)	11	3 (27)
Lutein/zeaxanthin	3	2 (67)	5	3 (60)	5	4 (80)
Total carotenoids	5	2 (40)	6	4 (67)	2	1 (50)
Vitamin C	1	1 (100)	10	6 (60)	18	14 (78)
Urinary potassium	1	1 (100	2	0	2	1 (50)
Flavonoids	–	–	1	0	8	6 (75)

McKinley noted that vitamin C and carotenoids were the most commonly measured biomarkers and that relatively few trials with mixed fruit and vegetables have used other biomarkers of fruit/vegetable consumption, for example, individual/total flavonoid status. She pointed to ongoing work involving novel use of panels of biomarkers, including plasma vitamin C, carotenoids, and possibly flavonoids to develop algorithms predictive of fruit and vegetable intake.^27^ This approach seeks to further explore the compositional complexities of fruit and vegetables. She concluded that a more global consideration of a panel of potential biomarkers is likely to be more useful than single compounds.

## References

[1] Black RE, Allen LH, Bhutta ZA (2008). Maternal and child undernutrition: global and regional exposures and health consequences. Lancet.

[2] Ramakrishnan U (2002). Prevalence of micronutrient malnutrition worldwide. Nutr. Rev.

[3] Garcia OP, Long KZ, Rosado JL (2009). Impact of micronutrient deficiencies on obesity. Nutr. Rev.

[b4] Global Strategy on Diet, Physical Activity and Health http://www.who.int/dietphysicalactivity/strategy/eb11344/strategy_english_web.pdf.

[5] Jehn M, Brewis A (2009). Paradoxical malnutrition in mother-child pairs: untangling the phenomenon of over- and under-nutrition in underdeveloped economies. Econ. Hum. Biol.

[6] Hooper L, Ashton K, Harvey LJ (2009). Assessing potential biomarkers of micronutrient status by using a systematic review methodology: methods. Am. J. Clin. Nutr.

[7] Raiten DJ, Namasté S, Brabin B (2011). Executive summary–Biomarkers of Nutrition for Development: Building a Consensus. Am. J. Clin. Nutr.

[8] Institute of Medicine (2010). Evaluation of Biomarkers and Surrogate Endpoints in Chronic Disease.

[9] Combs GF (2011). Determinants of selenium status in healthy adults. Nutr. J.

[10] Combs GF (2011). Differential Responses to selenomethionine supplementation by sex and genotype in healthy adults. Br. J. Nutr.

[11] Clark L (1996). The Nutritional Prevention of Cancer with Selenium 1983–1993: a Randomized Clinical Trial. J. Am. Med. Assoc.

[12] Klein EA (2011). Vitamin E and the risk of prostate cancer: the Selenium and Vitamin Cancer Prevention Trial (SELECT). J. Am. Med. Asoc.

[13] Bleys J, Navas-Acien A, Guallar E (2007). Serum selenium and diabetes in U.S. adults. Diabetes Care.

[14] Stranges S (2005). Effects of selenium supplementation on cardiovascular disease incidence and mortality: secondary analyses in a randomized clinical trial. Am. J. Epidemiol.

[15] Walston JD (2012). Sarcopenia in older adults. Curr. Opin. Rheumatol.

[16] Studenski S (2012). Conference Proceedings: Evidence-Based Criteria for Sarcopenia with Clinically Important Weakness. Semin Arthritis Rheum.

[17] Van Kan GA (2011). Sarcopenia: biomarkers and imaging (International Conference on Sarcopenia research). J. Nutr. Health Aging.

[18] Wagenknecht LE (1995). The insulin resistance atherosclerosis study (IRAS) objectives, design, and recruitment results. Ann. Epidemiol.

[19] American Diabetes Association (2010). Standards of medical care in diabetes. Diabetes Care.

[20] Noguchi Y (2006). Network analysis of plasma and tissue amino acids and the generation of an amino index for potential diagnostic use. Am. J. Clin. Nutr.

[21] Imaizumi A, Roessner MetabolomicsU (2012). Clinical Implementation of Metabolomics.

[22] Miyagi Y (2011). Plasma free amino acid profiling of five types of cancer patients and its application for early detection. PLoS ONE.

[23] Hisamatsu T (2012). Novel, objective, multivariate biomarkers composed of plasma amino acid profiles for the diagnosis and assessment of inflammatory bowel disease. PLoS ONE.

[24] Yamakado M (2012). Plasma amino acid profile is associated with visceral fat accumulation in obese Japanese subjects. Clin. Obesity.

[25] Jenab M (2009). Biomarkers in nutritional epidemiology: applications, needs and new horizons. Hum. Genet.

[26] Baldrick FR (2011). Biomarkers of fruit and vegetable intake in human intervention studies: a systematic review. Crit. Rev. Food. Sci. Nutr.

[27] Medical Research Council Research Portfolio http://www.mrc.ac.uk/ResearchPortfolio/Grant/Record.htm?GrantRef=G0901793&CaseId=16175.

